# Longitudinal Cluster Analysis of Hemodialysis Patients with COVID-19 in the Pre-Vaccination Era

**DOI:** 10.3390/life12111702

**Published:** 2022-10-26

**Authors:** Pasquale Esposito, Sara Garbarino, Daniela Fenoglio, Isabella Cama, Leda Cipriani, Cristina Campi, Alessia Parodi, Tiziana Vigo, Diego Franciotta, Tiziana Altosole, Fabrizio Grosjean, Francesca Viazzi, Gilberto Filaci, Michele Piana

**Affiliations:** 1 Department of Internal Medicine, University of Genoa, 16132 Genova, Italy; 2 Unit of Nephrology, Dialysis and Transplantation, IRCCS Ospedale Policlinico San Martino, 16132 Genova, Italy; 3 Dipartimento di Matematica (MIDA), Università di Genova, 16132 Genova, Italy; 4 Biotherapy Unit, IRCCS Ospedale Policlinico San Martino, 16132 Genova, Italy; 5 Department of Internal Medicine-Centre of Excellence for Biomedical Research, University of Genova, 16132 Genova, Italy; 6 IRCCS Ospedale Policlinico San Martino, 16132 Genova, Italy; 7 Unit of Nephrology, Dialysis and Transplantation, Fondazione IRCCS Policlinico San Matteo, 27100 Pavia, Italy; 8 Life Science Computational Laboratory (LISCOMP), IRCCS Ospedale Policlinico San Martino, 16132 Genova, Italy

**Keywords:** hemodialysis, COVID-19, cluster analysis, cytokines

## Abstract

**Simple Summary:**

Clinical presentation and outcomes of Coronavirus disease 2019 (COVID-19) are very heterogeneous. Among the different populations affected by COVID-19, special attention should be given to patients undergoing maintenance hemodialysis. Indeed, these patients present some peculiar characteristics that may influence disease course, leading to elevated morbidity and mortality. Furthermore, in hemodialysis patients, clinical presentations and disease severity may vary widely. Therefore, the identification of clinical and laboratory factors useful to stratify the risk of these patients could be of help in guiding clinical decision making. Starting from this observation, in this study, we tested and validated, in two cohorts of hemodialysis patients with COVID-19, an innovative analytical procedure that combines linear mixed effect modeling and cluster analysis on longitudinal data. The application of this strategy allowed patient stratification from simple and widely available data. Our results could contribute to improving COVID-19 management and supporting the implementation of longitudinal cluster analysis strategy in other clinical settings.

**Abstract:**

Coronavirus disease 2019 (COVID-19) in hemodialysis patients (HD) is characterized by heterogeneity of clinical presentation and outcomes. To stratify patients, we collected clinical and laboratory data in two cohorts of HD patients at COVID-19 diagnosis and during the following 4 weeks. Baseline and longitudinal values were used to build a linear mixed effect model (LME) and define different clusters. The development of the LME model in the derivation cohort of 17 HD patients (66.7 ± 12.3 years, eight males) allowed the characterization of two clusters (cl1 and cl2). Patients in cl1 presented a prevalence of females, higher lymphocyte count, and lower levels of lactate dehydrogenase, C-reactive protein, and CD8 + T memory stem cells as a possible result of a milder inflammation. Then, this model was tested in an independent validation cohort of 30 HD patients (73.3 ± 16.3 years, 16 males) assigned to cl1 or cl2 (16 and 14 patients, respectively). The cluster comparison confirmed that cl1 presented a milder form of COVID-19 associated with reduced disease activity, hospitalization, mortality rate, and oxygen requirement. Clustering analysis on longitudinal data allowed patient stratification and identification of the patients at high risk of complications. This strategy could be suitable in different clinical settings.

## 1. Introduction

The heterogeneity of clinical presentation and outcomes is a common feature of Coronavirus disease 2019 (COVID-19) [1]. This observation is also valid for maintenance hemodialysis (HD) patients, representing a distinct subgroup of COVID-19 patients [2,3]. Indeed, these patients have some features that may influence and differentiate disease course, including distinct renal failure etiologies, a high prevalence of comorbidities, frailty, and specific assets of the immune system. Moreover, the variability of dialysis programs and therapeutic regimens, such as logistical factors related to the organization of dialysis facilities, may significantly impact the dissemination and evolution of the infection in this setting [4]. Taking advantage of these peculiarities, we investigated the presence of different COVID-19 patient subtypes in this population using an innovative analytical procedure that combines linear mixed effect modeling and cluster analysis. Cluster analysis is an unsupervised learning algorithm that works by organizing patients into groups based on common characteristics [5]. This analysis aimed to stratify the patients, thus providing information to predict outcomes and address clinical management. Here, we present our experience with the application of cluster analysis on a small group of HD patients affected by COVID-19. We decided to consider only the first pandemic waves of 2020 to evaluate the natural history of the infection without potential confounding factors, such as vaccinations and antivirals.

## 2. Materials and Methods

### 2.1. Patients

For this study, we recruited 3-weekly maintenance hemodialysis patients with confirmed COVID-19 infection, from 16 March to 30 April 2020 (validation cohort) and from 1 October to 1 December 2020 (derivation cohort), enrolled at San Martino University Hospital of Genoa, Italy. To avoid selection bias, we enrolled consecutive patients in each period. Nasopharyngeal swabs for severe acute respiratory syndrome coronavirus-2 (SARS-CoV-2) were performed on HD patients presenting recent contact with COVID-19-positive patients and/or with fever or respiratory or gastrointestinal symptoms suspected of COVID-19. The diagnosis was confirmed by positive real-time reverse transcriptase (RT-PCR) assay for SARS-CoV-2. After the diagnosis, clinical management decisions were left to the attending physicians. Maintenance HD patients who tested negative for SARS-CoV-2 PCR constituted the control group.

### 2.2. Data Collection

We retrospectively collected clinical data, general laboratory, cytokine determinations, and cytofluorimetric analysis of activation status and detailed maturation of CD4+ and CD8+ lymphocyte subpopulations (Table S1) [6] at the time of COVID-19 diagnosis. Disease presentation severity was scored as 0 (asymptomatic patients), 1 (mildly symptomatic, i.e., only one symptom, including fever, cough, dysgeusia, or diarrhea), or 2 (fully symptomatic, i.e., fever + one additional symptom). In the derivation cohort, data were also collected in the first 4 weeks after the diagnosis (days 7, 14, 21, and 28), with each subject having at least two time points regarding the acquisition of each biomarker.

### 2.3. Cytokine Determinations

For cytokine determinations, blood samples collected in polypropylene tubes were centrifuged at 3200 rpm for 10 min. Plasma was separated by centrifugation and stored at −20 °C until assayed. Then, circulating cytokine levels were determined by an Ella automated Immunoassay platform (Protein Simple, Minneapolis, MN, USA).

### 2.4. Immunofluorescence Analyses

Immunofluorescence analyses were performed as previously described [7]. Prior to analysis, 100 µL of peripheral blood was incubated with specific fluorochrome-conjugated monoclonal antibodies (purchased from BD Biosciences), as indicated in Table S2. The samples were analyzed by a BD Fortessa X20 flow cytometer (BD Biosciences San Diego, CA, USA) using the BD FACS Diva™ software version 8.0 (BD Biosciences). Briefly, the differential expression of these markers allows the identification of six subsets in the human peripheral blood: naive (TN), stem cell memory (TSCM), central memory (TCM), transitional memory (TTM), effector memory (TEM), and terminal effector (TTE).

### 2.5. General Statistical Methods

Data are presented as mean ± standard deviation (SD), or interquartile ranges (IQR) if not normally distributed (evaluated by Shapiro test). A Mann–Whitney test was used to assess the differences among patients affected by COVID-19 and the control group, and among patients of different clusters. Proportions for categorical variables were compared using Fisher’s test. A two-tailed *p* value < 0.05 was considered statistically significant.

### 2.6. Statistical Methods for Clustering Analysis

To investigate the presence of different COVID-19 HD subtypes, we used an innovative model that combines linear mixed effect modeling and cluster analysis. The model was implemented in R (https://www.rstudio.com/ (accessed on 11 May 2022)). First, in the derivation cohort, we performed a feature reduction procedure by selecting the 10 features whose distribution showed, at the same time, the highest intergroup difference (COVID-19 HD and HD without COVID-19) and the lowest intragroup variability using a Mann–Whitney test [8], and for which at least 90% of COVID-19 HD patients had a baseline acquisition. Then, we used a linear mixed effect model (LME) [9,10] with two fixed and two random effects (both slope and intercept) on the COVID-19 HD longitudinal data. For each individual and each of the 10 selected features, the estimated fixed and random effects describe the feature progression on the overall cohort (the fixed effect) and the individual variation (the random effect). Then, to identify potential different clusters of COVID-19 HD patients, we performed a k-means clustering analysis [11] using as the input the 20 features returned by the LME model (slope and intercept for each of the 10 features) and distinguish the different clusters, following a silhouette analysis with Dice distance.

Finally, the most significant clinical and laboratory parameters discriminating patients of the derivation cohort were used in a validation cohort to compute the Euclidean distances between each validation subject and the cluster centroids to assign each patient to different clusters, which are compared in the following section.

## 3. Results

### 3.1. Longitudinal Clustering of the Derivation Cohort

**Figure 1:**
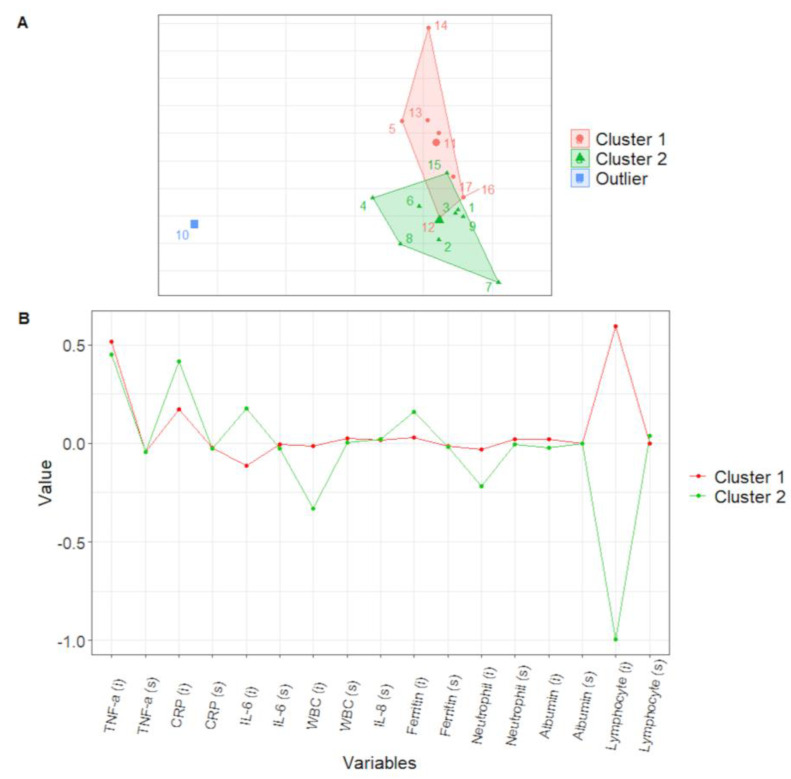
Cluster analysis of longitudinal data performed on the derivation cohort. Top panel (**A**): result of cluster analysis. Bottom panel (**B**): centroid profiles of Clusters 1 and 2. Abbreviations: white blood cell count, WBC; C-reactive protein, CRP; interleukin, IL; tumor necrosis factor-alfa, TNF-α; intercept, i; slope, s.

The derivation cohort (DC) constituted 17 HD patients (66.7 ± 12.3 years, eight males) with a molecular diagnosis of COVID-19 during the second pandemic wave of 2020 (October–December). In this cohort, overall, we collected 51 longitudinal observations over the four time points (days 7, 14, 21, and 28) on the 17 subjects. First, we performed a feature reduction procedure. Specifically, the baseline values of COVID-19 HD were compared with a sex–age-matched control group of six HD patients (70.0 ± 9.4 years, three males) (Table S3). As explained in detail in the Materials and Methods section, according to the baseline evaluation, we selected the 10 most significant variables to build the cluster analysis model. These variables included: C-reactive protein (CRP), white blood cell, neutrophil, and lymphocyte counts; albumin and ferritin serum levels; and interleukin (IL)-1β, IL-8, IL-6, and tumor necrosis factor (TNF)-α circulating levels. Then, we performed clustering analysis using an LME on the longitudinal data of all 17 patients of the DC (Figure S1 shows the individual LME fits). This analysis returned two well-balanced (seven vs. nine patients) clusters (Cluster 1 and Cluster 2, respectively, Figure 1A) and one cluster consisting of one subject, which was excluded from further analysis. The centroid profiles of Clusters 1 and 2 (Figure 1B) showed a significant difference in the value of lymphocyte count (*p* = 0.012). The comparison between the two clusters showed that within Cluster 1, there were more female patients (*p* = 0.01) presenting significantly higher lymphocyte counts (Cluster 1, 1.1 ± 0.5 vs. Cluster 2, 0.4 ± 0.2 x109/L; *p* = 0.012) and lower lactate dehydrogenase (LDH), CRP (Cluster 1, 22.2 ± 16.3 vs. Cluster 2, 72.6 ± 44.1 mg/dL; *p* = 0.017), and CD8 + T memory stem cells (Cluster 1, 0.6 ± 0.4 vs. Cluster 2, 2.3 ± 1.7%; *p* = 0.038, Figure S2) (Table 1).

### 3.2. Validation Cohort

We then tested our clustering strategy for predicting clinical outcomes in a validation cohort (VC), constituted of 30 HD patients (73.3 ± 16.3 years, 16 males) affected by COVID-19 during the first pandemic wave of 2020 (March–April).

According to those found in DC, we selected the most significant clinical and laboratory parameters that could be used to discriminated between the two clusters.

These factors included sex, age, neutrophil and lymphocyte percentages, CRP, procalcitonin, and LDH levels. CD8 + T memory stem cell count was not included at this stage because this analysis is not widely available.

As a standard analysis in cluster validation, and as described in the Materials and Methods section, we used these variables to assign each new patient to Cluster 1 or 2. Hence, in the VC, Cluster 1 or 2 constituted 16 and 14 subjects, respectively. The two clusters were comparable for general characteristics, even though in Cluster 1 there was a higher prevalence of female patients. Moreover, within Cluster 1, both biochemical profile and disease severity presentation were significantly milder than in Cluster 2 (clinical severity score distribution Cluster 1 vs. Cluster 2, *p* = 0.018). Similarly, we found significant differences in patient outcomes. In particular, in Cluster 1, three patients required hospitalization (18%), one patient died (6.5%), and none required high-flow oxygen therapy, whereas, in Cluster 2, nine patients were hospitalized (64%), five died (35%), and four patients (28%) required high-flow oxygen supply (*p* = 0.02, *p* = 0.07, and *p* = 0.02, respectively). Finally, there were no significant differences in the duration of the infection (Table 2). The positive predictive values (PPV) and negative predictive values (NPV) associated with key clinical parameters are shown in Table 3.

## 4. Discussion

In this study, we found that cluster analysis on longitudinal data provided information about the differentiation of baseline characteristics and overall disease progression in HD patients with COVID-19. In particular, the application of this analysis strategy in our DC resulted in the identification of two distinct clusters: Cluster 1 and Cluster 2. The comparison of these clusters showed that patients included in Cluster1 presented a better biochemical profile, as proven by a lower incidence of lymphopenia and lower levels of inflammatory and immune activation markers, such as CRP and CD8 + T memory stem cells.

Interestingly, within Cluster 1, there was also a higher prevalence of female patients, who, according to data from several studies, often develop a less severe form of COVID-19 [12]. Moreover, when the model developed on the DC was tested in the VC, we found confirmation that biochemical differences between the two clusters had relevant clinical implications, as also shown by the calculation of the predictive values. Thus, we confirmed that the patients included in Cluster 1 had milder disease severity and better outcomes in terms of rate of hospitalization, death, and oxygen requirement.

Regarding the inflammatory parameters, the analysis of serum albumin levels deserves special consideration. It is well known that serum albumin concentration is negatively influenced by acute diseases, and in COVID-19 patients, low albumin levels have been related to poor prognosis [13]. In our cohort, we observed that while albumin level was lower in COVID-19 patients than in controls, no differences were found when comparing the patients assigned to the different clusters.

Possible explanations for these findings may be that inflammation is not the only determinant of serum albumin, and nutritional status and dialysis adequacy may influence it [14]. Therefore, COVID-19 might not have impacted all these aspects. However, it cannot be ruled out that these data may result from the small size of our cohort.

Cluster analysis has been already applied to the general population of patients affected by COVID-19 in different clinical contexts. San-Cristobal R et al. used this methodology to predict disease severity within 72 h of hospital admission in patients with confirmed COVID-19 [15]. By collecting routine clinical and laboratory variables, the authors were able to describe three distinct clusters that showed a strong association with different clinical outcomes. Similar results were also observed in larger studies where more complex and multiple factors, including clinical, biochemical, and immune parameters, were considered. Additionally in these cases, cluster analysis resulted in a relevant capacity to discriminate distinct patient profiles associated with different clinical presentations and outcomes [16,17,18]. Interestingly, some authors found that among complex parameters, simple indicators, such as standard laboratory examinations (CRP, LDH, etc.), and symptoms, such as fever, fatigue, as well as, nausea and vomiting, may have a role in indicating the disease severity and prognosis [16].

Beyond clinical studies, analytical models based on cluster analysis in the setting of COVID-19 have also been used in the interpretation of big data collected at both a national and international level, taking advantage of methods developed in computer science and machine learning. In these cases, cluster analysis was extended to evaluate health system performances and the social and economic impact of the pandemic [19,20,21]. Overall, these studies, despite different patient populations and designs, have shown the feasibility and utility of cluster analysis in the management of data recorded during the COVID-19 pandemic. In addition to that already published, here we report the first example of the application of cluster analysis on longitudinal data collected from COVID-19 patients undergoing maintenance HD. The rationale for testing this strategy in this population is that HD patients constitute a peculiar subgroup of COVID-19 patients. Indeed, these patients present some distinct characteristics, including a high prevalence of comorbidity, specific assets of the immune system, and factors related to the organization of dialysis facilities that make them susceptible to severe infections [22,23]. COVID-19 was not an exception, and many reports have shown that HD patients were burdened by significantly higher morbidity and mortality when compared with the general population [24,25].

However, in HD patients, clinical presentation and severity of COVID-19 may vary widely. Therefore, the characterization of clinical and laboratory factors useful to stratify these patients could help the clinical decision process. Our results show that this is possible using cluster analysis, which may be functional in identifying the patients at high risk of complications deserving special attention. Most importantly, this methodology, by the initial analysis of a complex dataset, allowed us to build a final predictive model based on simple clinical and laboratory parameters.

We are aware of the limitations of this study, mainly due to the small number of subjects evaluated that probably influenced our ability to discriminate laboratory and clinical differences between the clusters. On the other hand, this aspect could be partially compensated by the fact that we focused our analysis on a specific and well-characterized population. Moreover, we know that COVID-19 is a rapidly evolving disease and, as for the general population, including HD patients, the introduction of vaccinations and antiviral drugs and the emergence of new virus variants have significantly changed the natural history of the disease [26]. Thus, conceivably, the model presented here is not directly applicable in the current context. Nevertheless, we think that beyond rough data, our findings prove the effectiveness of a methodology that may have a wide application.

In particular, the implementation of cluster analysis may be valuable, both for scientific and clinical purposes. Indeed, it may reveal hidden associations among laboratory, instrumental, demographic, and clinical factors, thus improving our understanding of disease pathophysiology, such as inflammation in COVID-19 patients. On the other hand, the capacity to assign patients to “low-risk” and “high-risk” categories may influence clinical management and therapeutic choices. Finally, all these effects may assist in the allocation of resources [27].

## 5. Conclusions

Cluster analysis on longitudinal data was effective at stratifying HD patients with COVID-19 starting from the analysis of a few laboratory and clinical variables. These findings support the application of this technique as a suitable tool to translate research findings into clinical practice, possibly in large cohorts, not only in the management of COVID-19, but also in other clinical settings.

## Figures and Tables

**Table 1:** Baseline characteristics of the derivation cohort of COVID-19-positive HD patients according to cluster assignment.

	Cluster 1	Cluster 2	*p*
N	7	8 *	
Age, years	68.1 ± 15.6	64.4 ± 10.7	0.7
Sex, M/F	1/6	7/1	0.01
Dialysis vintage, months	32.6 ± 19.6	72.8 ± 56.3	0.1
WBC, ×10^9^/L	5.4 ± 1.3	4.4 ± 1.5	0.33
Lymphocytes, ×10^9^/L	1.1 ± 0.5	0.4 ± 0.2	0.012
Neutrophils, ×10^9^/L	3.8 ± 1.4	3.5 ± 1.5	1
Lymphocytes, (% WBC)	21.0 ± 7.5	11.8 ± 4.9	0.03
Neutrophils, (% WBC)	66.6 ± 13.8	78.6 ± 6.2	0.06
CD8 + TSCM/CD8+	0.6 ± 0.4	2.3 ± 1.7	0.038
Albumin, g/L	34.6 ± 3.5	33.1 ± 4.2	0.6
LDH, U/L	201.2 ± 42.3	323.0 ± 88.8	0.012
Procalcitonin, ng/ml	0.9 ± 0.5	3.3 ± 3.6	0.02
CRP, mg/L	22.2 ± 16.3	72.6 ± 44.1	0.017

* Baseline data were available for 8/9 COVID-19 HD patients included in Cluster 2. Abbreviations: hemodialysis, HD; white blood cells, WBC; C-reactive protein, CRP; lactate dehydrogenase, LDH; T memory stem cells, TSCM.

**Table 2:** Validation cohort of cluster analysis performed using COVID-19-positive HD patients: General characteristic and comparison between patients assigned to different clusters.

	All Patients	Cluster 1	Cluster 2	*p* Cluster 1 vs. 2
N	30	16	14	
Age, years	73.3 ± 16.3	75.7 ± 15.5	70.4 ± 17.3	0.4
Sex, M/F (M%)	16/14 (53)	6/10 (37)	10/4 (71)	0.08
Dialysis vintage, months	36 (14–71)	43 (28–75)	27 (7–64)	0.1
WBC, ×10^9^/L	6.5 ± 4.9	5.4 ±5.1	7.7± 4.6	0.02
Lymphocytes, ×10^9^/L	0.7 ± 0.3	0.9± 0.3	0.6 ± 0.3	0.03
Neutrophils, ×10^9^/L	5.0 ± 4.3	3.6 ± 3.9	6.6± 4.4	0.01
Lymphocytes, (% WBC)	15.4 ± 9.5	21.2 ± 9.5	9.1 ± 4.3	<0.0001
Neutrophils, (% WBC)	70.1 ± 19.7	59.3 ± 20.8	82.5 ± 6.9	<0.0001
LDH, U/L	234.0 ± 72.1	207.9 ± 49.1	260.1 ± 83.4	0.09
Albumin, g/L	33.0 ±4 2	33.9 ± 2	32.8 ± 2.2	0.3
Procalcitonin, ng/ml	1.4 (0.6–4.6)	0.8 (0.4–1.6)	3.6 (1.2–9.5)	0.07
CRP, mg/L	14.7 (4.0–39.0)	8.0 (2.8–20.1)	32.6 (12.1–50.1)	0.01
Clinical severity score > 0, n (%)	27 (90)	13 (80)	14 (100)	0.04
Clinical severity score, n - 0 - 1 - 2	3 20 7	3 12 1	0 8 6	0.018
Outcomes				
Hospitalization, n (%)	12 (40)	3 (18)	9 (64)	0.02
Death, n (%)	6 (20)	1 (6.2)	5 (35)	0.07
High-flow oxygen therapy, n (%)	4 (13)	0	4 (28)	0.02
Duration of SARS-CoV-2 infection, days	21 (13–28)	22 (14.5–35)	20 (11–31)	0.38

Abbreviations: hemodialysis, HD; white blood cells, WBC; C-reactive protein, CRP; lactate dehydrogenase, LDH.

**Table 3:** The positive predictive values (PPV) and negative predictive values (NPV) associated with the key clinical parameters in the validation cohort.

	NPV	PPV
Clinical severity score > 0	0.19 (3/16)	1 (14/14)
Hospitalization	0.81 (13/16)	0.64 (9/14)
Death	0.94 (15/16)	0.36 (5/14)
High-flow oxygen therapy	1 (16/16)	0.29 (4/14)

## Data Availability

The data underlying this article are available in Harvard Dataverse, at https://doi.org/10.7910/DVN/KDKR4V (accessed on 16 September 2022).

## Supplementary Materials

The following supporting information can be downloaded at: https://www.mdpi.com/article/10.3390/life12111702/s1. Figure S1: Individual linear mixed model fits. Figure S2: Representative examples of the analysis strategy of CD8 + T cell differentiation. Table S1: Full dataset of baseline clinical and laboratory parameters collected from COVID-19-positive and COVID-19-negative HD patients. Table S2: Reagents used for immunofluorescence analyses. Table S3: Comparisons between baseline characteristics of COVID-19-positive and COVID-19-negative HD patients.
