# Supplementary material for: Longitudinal Cluster Analysis of Hemodialysis Patients with COVID-19 in the Pre-Vaccination Era
Source: Life (Basel). 2022 Oct 26;12(11):1702. doi: 10.3390/life12111702 (PMC9695171; doi:10.3390/life12111702)
Supplement: Supplementary file 1 [file life-12-01702-s001.zip › life-1965706-supplementary.pdf]

Supplementary Materials for Longitudinal Cluster Analysis of Hemodialysis Patients with COVID-19 in the Pre-Vaccination Era

Table S1. Full dataset of baseline clinical and laboratory parameters collected from COVID-19 positive and COVID-19 negative HD patients.

Clinical Parameters	Laboratory Parameters (On Blood)	Circulating Cytokines Levels	Cytometric Analysis: Profiles of CD4+ T Helper (Th)	Cytometric Analysis: Maturation Status of CD8+ and CD4+ T cells	Cytometric Analysis: Activation Status of CD8+ and CD4+ T Cells
Age	Serum creatinine	IL-1β	Tfh/CD4+	Naïve CD45RA+CCR7+CD95-/CD8+	CD38+DR+/CD8+
Sex	BUN	TNF-α	Tfh CCR4+/CD4+	CD8+TSCM/CD8+	CD38+DR+/CD4+
Dialysis vintage	WBC	IL-6	Tfh CXCR3+/CD4+	CM CD8+: CD45RA-CCR7+CD28+/CD8+	
Diabetes	Neutrophil count	IL-8	Tfh CCR6+/CD4	TM CD8+: CD45RA-CCR7-CD28+/CD8+	
CVD disease	Lymphocyte count		CD161+CCR6+/CD4+	EM CD8+: CD45RA-CCR7-CD28-/CD8+	
COPD/asthma	CRP		Th1 CCR6-CCR4-CXCR3+/CD4+	TE CD8+: CD45RA+CCR7-CD28-/CD8+	
Clinical severity*	Procalcitonin,		Th2CCR6-CCR4+CXCR3-/CD4+	Naïve CD45RA+CCR7+CD95-/CD4+	
	Ferritin		Th17CCR6+CCR4+CXCR3-/CD4+	CD4+TSCM/CD4+	
	LDH		Th17CCR6+CCR4+CXCR3-CD161+/CD4+	CM CD4+: CD45RA-CCR7+CD28+/CD4+	
	Albumin		Th1-Th17 CCR6+CCR4-CXCR3+/CD4+	TM CD4+: CD45RA-CCR7-CD28+/CD4+	
	D-Dimer		Th1-Th17 CCR6+CCR4-CXCR3+CD161+/CD4+	EM CD4+: CD45RA-CCR7-CD28-/CD4+	
			Th9CCR4-CCR6+/CD4+	TE CD4+: CD45RA+CCR7-CD28-/CD4+	
			Th22CCR10+CCR4+CCR6+/CD4+		

Abbreviations: hemodialysis, HD; cardiovascular disease, CVD; chronic obstructive pulmonary disease, COPD; white blood cell count, WBC; C-reactive protein, CRP; lactate dehydrogenase, LDH; interleukin, IL; *Tumor necrosis factor-alfa,* TNF-α; cluster of differentiation, CD; central memory, CM; transitional memory, TM; effector memory, EM; terminal effector, TE; T-memory stem cells, TSCM. * Clinical presentation severity was scored as reported in the method section of the paper.

Table S2. Reagents used for immunofluorescence analyses.

Type of Marker	Specificity	Clone	Fluorochrome
Cell lineage	CD3	UCHT1	BV786
	CD4	RPA-T4	APC-H7
	CD8	RPA-T8	PE-Cy7
Maturation stage	CD45RA	H100	BV605
	CD197(CCR7)	150503	PE-CF594
	CD95	DX2	BV711
	CD27	M-T271	BB515
Th subsets	CXCR5	RF8B2	BV650
	CD196(CCR6)	11A9	BV421
	CCR10	1B5	PerCP.Cy5,5
	CCR4 (CD194)	1G1	PE
	CD183(CXCR3)	1C6	PE-Cy7
	CD161	DX12	APC
Activation	DR	G46-6	PerCP.Cy5,5
	CD38	HiT2	PE

Abbreviations: PE—phycoerythrin; Cy—cyanine; BV—brilliant violet; FITC—fluorescein; APC—Allophycocyanin; PerCP—Peridinin-chlorophyll proteins; BB—brilliant blue.

Table S3. Comparisons between baseline characteristics of COVID-19 positive and COVID-19 negative HD patients.

	COVID-19 Positive HD	COVID-19 Negative HD	*p*
**N**	16*	6	
**Age, years**	66.7 ± 12.3	70.0 ± 9.4	0.6
**Sex, M/F**	8/8	3/3	1
**Dialysis vintage, months**	51.5 ± 46.0	46.3 ± 26.5	0.6
**Diabetes, N (%)**	5 (31)	3 (50)	0.6
**CVD disease, N (%)**	8 (50)	4 (66)	0.6
**COPD/asthma, N (%)**	2 (12.5%)	0	1
**WBC, ×10^9/L**	4.9 ± 1.4	7.3 ± 1.9	0.007
**Lymphocytes, ×10^9/L**	0.7 ± 0.4	1.1 ± 0.3	0.07
**Neutrophils, ×10^9/L**	3.7 ± 1.4	5.6 ± 1.7	0.03
**Lymphocytes, (% WBC)**	73.6 ± 12.0	76.1 ± 5.4	0.8
**Neutrophils, (% WBC)**	16.0±7.6	14.7±3.4	0.9
**CRP, mg/L**	39.8 (27.0-66.3)	4.5 (3.0-11.7)	0.006
**Ferritin, μg/L**	812 (591-1236)	284 (227-408)	0.02
**D-dimer, μg/L**	4731±11183	739±1330	0.04
**Albumin, g/L**	34±4	37±2	0.05
**TNF-α, pg/ml**	48.0±16.0	22.9±4.0	<0.001
**IL-6, pg/ml**	22.8 (15.1-40.2)	5.2 (3.3-8.3)	0.006
**IL-8, pg/ml**	52.5 (23.6-71.3)	20.2 (9.0-32.0)	0.02
**IL-1b, pg/ml**	1.1±0.9	0.4±0.0	0.009
**ThfCCR4+/CD4+**	0.3±0.2	1.9±1.4	0.04
**Th2CCR6-CCR4+CXCR3-/CD4+**	1.8±0.8	4.6±2.2	0.02
**Th17CCR6+CCR4+CXCR3-/CD4+**	1.7±1.1	3.6±0.9	0.003
**Th9CCR4-CCR6+/CD4+**	21.2±5.4	29.5±5.3	0.01
**Th22CCR10+CCR4+CCR6+/CD4+**	0.01±0.01	0.04±0.03	0.06
**Th17CCR6+CCR4+CD161+/CD4+**	1.4±1.0	2.8±1.0	0.02
**CD38+DR+/CD8**	16.0±9.1	6.9±4.6	0.02
**CD8+TSCM/CD8+**	1.7±1.5	0.7±0.5	0.04

Data are expressed by mean ± standard deviation (SD) or median-interquartile ranges (IQR) if they were not normally distributed. * Baseline data were available in 16/17 COVID-19 HD patients enrolled in this study. Abbreviations: hemodialysis, HD; cardiovascular disease, CVD; chronic obstructive pulmonary disease, COPD; white blood cell count, WBC; C-reactive protein, CRP; lactate dehydrogenase, LDH; interleukin, IL; *Tumor necrosis factor-alfa,* TNF-*α*; T helper, Th; T-memory stem cells, TSCM.

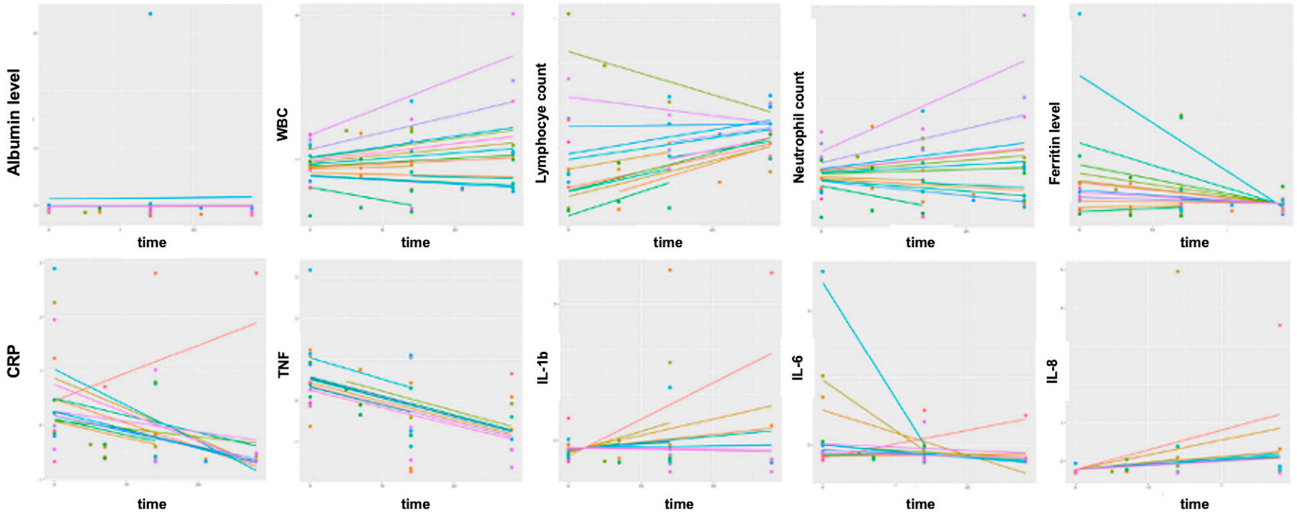

Figure S1. Individual linear mixed model fits. Each plot represents the individual LME fits estimated by the model for a specific feature of interest. In each plot, each line represents the fit of a specific COVID-19 HD subject, and colours are consistent among plots. Abbreviations: white blood cell count, WBC; C-reactive protein, CRP; interleukin, IL; *Tumor necrosis factor-alfa*, TNF.

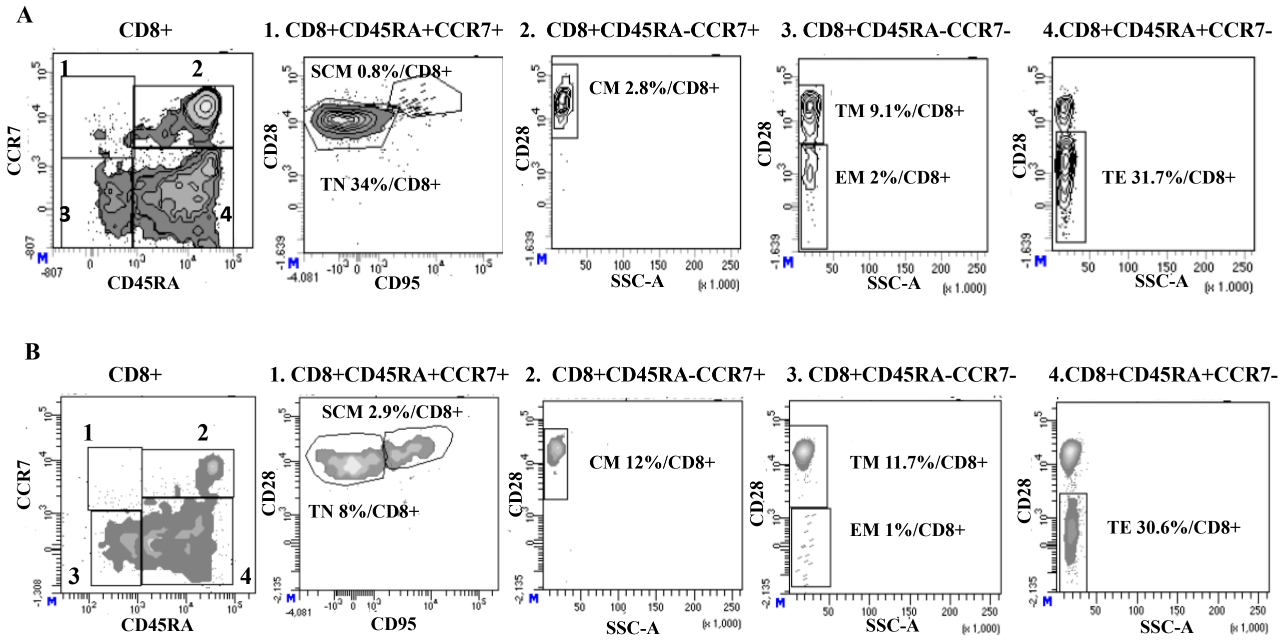

Figure S2. Representative examples of the analysis strategy of CD8+T cell differentiation. Panel A and B show the dynamic of peripheral CD8+ T cell maturation in patient 11 (Cluster 1) and in patient 6 (Cluster2) by differential expression of CD45RA, CCR7, CD28 and CD95 markers. TN: true naïve; SCM: stem cell memory; TM: transitional memory; EM: effector memory; TE: terminal effector.
